# Supplementary material for: Make or break: The influence of expected challenges and rewards on the motivation and experience associated with cognitive effort exertion
Source: bioRxiv. 2023 Dec 7:2023.12.05.570154. Preprint. [Version 1] doi: 10.1101/2023.12.05.570154 (PMC10723292; doi:10.1101/2023.12.05.570154)
Supplement: Supplementary file 1 [file media-1.pdf]

Supplement Results

Congruency Effects (Supplement Table 1& Supplement Table 2)

We examined the effect of current trial congruency (i.e., word and ink color matches) and previous trial congruency on task performance. There were significant main effects for both current trial congruency and previous trial congruency on trial-wise reaction time and accuracy. Specifically, current trial congruency as well as previous incongruent trial led to faster and more accurate performance. We did not see any interactions with either challenge level (Experiment 1) or reward level (Experiment 2), suggesting that these factors were not influencing conflict adaptation.

Supplement Table 1. Mixed Model Results for Reaction Time and Accuracy Based on Current and Previous Trial Congruency (Experiment 1)

Predictors	Trial-wise Reaction Time			Accuracy		
	Estimates	*SE*	*p-value*	*Log Odds Ratios*	*SE*	*p-value*
Current Trial Congruency	-94.24	28.80	**< 0.001*** **	0.34	0.14	**0.012***
Prev Trial Congruency	19.29	8.12	**0.018***	-0.22	0.11	**0.040***
Hard - Easy	-23.09	5.46	**<0.001*****	0.17	0.08	**0.030***
Interval Goal Completion	-149.90	10.73	**<0.001*****	1.83	0.11	**<0.001*****
Scaled Trial Session Num	57.00	13.52	**<0.001*****	-0.35	0.13	**0.006****
Scaled Interval Session Num	-54.99	13.07	**<0.001*****	0.26	0.12	**0.037***
Scaled Interval Block Num	-4.35	2.60	0.094	0.06	0.04	0.107
Current * Prev Congruency	-12.10	11.00	0.271	0.21	0.16	0.203
Current Congruency * Challenge	4.20	6.93	0.545	0.04	0.04	0.723
Previous Congruency * Challenge	-2.20	6.97	0.752	-0.01	0.10	0.924

Supplement Table 2. Mixed Model Results for Reaction Time and Accuracy Based on Current and Previous Trial Congruency (Experiment 2)

Predictors	Trial-wise Reaction Time			Accuracy		
	Estimates	*SE*	*p-value*	*Log Odds Ratios*	*SE*	*p-value*
Current Trial Congruency	-115.39	7.32	**< 0.001*****	0.34	0.07	**<0.001*****
Prev Trial Congruency	1.80	3.72	0.628	-0.01	0.05	0.782

High Reward – Low Reward	-4.43	2.77	0.109	0.00	0.04	0.955
Hard - Easy	-27.83	2.92	**<0.001***	0.26	0.02	**<0.001***
Interval Goal Completion	-159.41	4.69	**<0.001***	1.74	0.05	**<0.001***
Scaled Trial Session Num	-26.78	8.06	**0.001***	-0.53	0.11	**<0.001***
Scaled Interval Session Num	15.60	7.86	**0.047***	0.52	0.11	**<0.001***
Scaled Interval Block Num	-1.04	1.23	0.398	0.01	0.02	0.547
Current * Prev Congruency	-7.55	5.34	0.158	-0.13	0.08	0.109
Current Congruency * Reward	-0.69	3.39	0.838	0.04	0.05	0.406
Previous Congruency * Reward	-0.60	3.39	0.859	0.01	0.05	0.871

Experiment 1 Supplement

Supplement Table 3. Mixed Model Results for Reaction Time and Accuracy (Experiment 1)

Predictors	Reaction Time (for accurate trials)			Accuracy		
	Estimates	*SE*	*p-value*	*Log Odds Ratios*	*SE*	*p-value*
Hard - Easy	-36.48	6.08	< **0.001***	0.22	0.07	**<0.001***
Trial Goal Completion[1]	-42.95	10.40	< **0.001***	-0.00	0.10	0.972
Interval Goal Completion	-203.50	22.35	< **0.001***	1.77	0.65	**<0.001***
Scaled Trial Session Num[2]	37.00	14.97	**0.013***	-0.39	0.11	**0.010****
Scaled Interval Session Num	-38.08	14.42	**0.008***	0.29	0.18	**0.046***
Scaled Interval Block Num	-1.89	2.81	0.501	0.03	0.03	0.370
Challenge X Trial Goal Completion	11.79	6.72	*0.079*	-0.22	0.07	**0.016***

1. Whether the trial was completed before or after reaching the goal (0: before; 1: after)
2. Scaled trial number across the experiment session

Effects of affective experience and performance on subsequent task performance

Because the task was organized into blocks of intervals, we were also able to examine whether the previous interval carried-over impact to the current interval. Specifically, we investigated whether affective experiences and performance tied to the previous interval influenced the current interval's affect and performance. For these analyses, we selected only

intervals where the goal had been attained for the current interval and controlled for challenge level.

We found that participants who reported higher stress in the previous interval were slower to respond correctly on the current interval ($F(1, 9.17) = 4.11$, $p = 0.043$). We did not find any other significant relationships between affective experiences during the previous interval (stress or positive affect) and performance on the current interval (Supplement Table 4, Supplement Table 5), nor did we find significant relationships between performance on the previous interval and affect during the current interval (Supplement Table 6).

We did, however, find a relationship between performance on the previous and current intervals. Specifically, after successfully meeting their goal on one interval, participants completed more correct trials ($F(1, 118.4) = 9.72$, $p = 0.002$) and were faster in performing correct trials ($F(1, 28.6) = 12.71$, $p < 0.001$) in the next interval (Supplement Table 7). While intriguing, we hasten to note that this final carry-over effect on its own could reflect autocorrelation due to variability in attention and arousal over the course of the session.

Supplement Table 4. Mixed Model Results for Response Rate and Mean Accurate RT Based on Previous Interval's Stress Rating (Experiment 1)

Predictors	Response Rate			Mean Accurate RT		
	Estimates	*SE*	*p-value*	*Estimates*	*SE*	*p-value*
Previous Stress	-0.00	0.00	0.483	5.76	2.84	**0.043***
Hard - Easy	0.06	0.01	**< 0.001*****	-34.91	6.48	**< 0.001*****
Average Congruency	-0.00	0.01	0.787	-6.12	4.42	0.166
Scaled Interval Session Num	0.00	0.01	0.789	1.86	4.25	0.662
Scaled Interval Block Num	0.01	0.01	0.489	6.69	5.00	0.181

Supplement Table 5. Mixed Model Results for Response Rate and Mean Accurate RT Based on Previous Interval's Positive Affect Rating (Experiment 1)

Predictors	Response Rate			Mean Accurate RT		
	Estimates	*SE*	*p-value*	*Estimates*	*SE*	*p-value*
Previous Positive Affect	0.01	0.00	0.154	-2.56	2.51	0.308
Hard - Easy	0.05	0.01	**< 0.001********	-34.44	7.34	**< 0.001*****
Average Congruency	0.02	0.01	0.090	-5.99	5.45	0.272
Scaled Interval Session Num	-0.01	0.01	0.408	-3.45	4.95	0.485
Scaled Interval Block Num	0.00	0.01	0.854	-1.53	5.28	0.772

Supplement Table 6. Mixed Model Results for Affective Ratings Based on Previous Interval's Goal Completion (Experiment 1)

Predictors	Stress			Positive Affect		
	Estimates	*SE*	*p-value*	*Estimates*	*SE*	*p-value*
Previous Interval Goal Completion	-0.47	0.36	0.195	-0.31	0.41	0.452
Hard - Easy	0.40	0.13	**0.002****	-0.21	0.10	**0.035***
Average Congruency	0.10	0.09	0.249	-0.09	0.11	0.402
Scaled Interval Session Num	-0.04	0.09	0.641	-0.27	0.10	**0.007****
Scaled Interval Block Num	-0.02	0.10	0.809	-0.03	0.11	0.819

Supplement Table 7. Mixed Model Results for Response Rate and Mean Accurate RT Based on Previous Interval's Goal Completion (Experiment 1)

Predictors	Response Rate			Mean Accurate RT		
	Estimates	*SE*	*p-value*	*Estimates*	*SE*	*p-value*
Previous Interval Goal Completion	0.08	0.02	**0.002****	-56.69	15.90	**< 0.001*****
Hard - Easy	0.05	0.01	**< 0.001*****	-36.60	6.91	**< 0.001*****
Average Congruency	0.01	0.01	0.418	-5.20	3.47	0.134
Scaled Interval Session Num	-0.00	0.01	0.431	1.58	3.38	0.640
Scaled Interval Block Num	-0.00	0.01	0.901	2.36	3.65	0.517

Experiment 2 Supplement

Supplement Table 8. Mixed Model Results for Interval Goal Attainment and Higher Goal Attainment Based on Challenge and Reward Level (Experiment 2)

Predictors	Interval Goal Reached			High Goal Reached		
	Log Odds Ratios	*SE*	*p-value*	*Log Odds Ratios*	*SE*	*p-value*
Hard - Easy	-1.52	0.16	**< 0.001*** **	0.22	0.05	**< 0.001*** **
High Reward – Low Reward	0.00	0.09	0.983	0.10	0.04	**0.019***
Average Congruency	0.07	0.05	0.150	0.10	0.04	**0.011***
Scaled Interval Session Num	0.17	0.05	**0.001*** **	0.17	0.04	**< 0.001*** **
Scaled Interval Block Num	0.02	0.05	0.676	0.05	0.04	0.167
Challenge X Reward	0.06	0.07	0.396	-0.05	0.04	0.179

Supplement Table 9. Mixed Model Results for Reaction Time and Accuracy based on Challenge and Reward Level (Experiment 2)

Predictors	Response Time (for accurate trials)			Accuracy		
	Estimates	*SE*	*p-value*	*Log Odds Ratios*	*SE*	*p-value*
Hard - Easy	-37.03	3.92	**< 0.001*** **	0.30	0.04	**< 0.001*** **
High Reward – Low Reward	-4.60	1.60	**0.004****	0.01	0.02	0.635
Trial Goal Completion	-34.22	5.48	**< 0.001*** **	-0.18	0.04	**<0.001*** **
Interval Goal Completion	-171.30	15.89	**< 0.001*** **	1.83	0.46	**<0.001*** **
Scaled Trial Session Num	-50.73	8.65	**< 0.001*** **	-0.63	0.06	**<0.001*** **
Scaled Interval Session Num	38.68	8.40	**< 0.001*** **	0.61	0.21	**<0.001*** **
Scaled Interval Block Num	-2.18	1.33	0.101	0.01	0.02	0.599
Challenge X Reward	1.00	1.57	0.526	-0.02	0.02	0.291
Challenge X Trial Goal Completion	9.12	3.41	**0.007****	-0.12	0.04	**0.011***
Reward X Trial Goal Completion	-1.23	3.28	0.707	0.03	0.05	0.474

(Continued on next page)

Supplement Table 10. Summary of key results across experiments

Predictors	Response Rate	Trial-wise Reaction Time (Accurate Trials)	Trial-wise Accuracy	Stress Rating[1]	Positive Affect Rating[1]
	Beta Estimate	Beta Estimate	Log Odds Ratio	Beta Estimate	Beta Estimate
**Experiment 1: Challenge level**					
Interval Goal Completion	**0.42*** **	**-203.50*** **	**1.77*** **	**-1.71*** **	**2.33*** **
Trial Goal Completion[1]	**-**	**-40.21*** **	-0.01	**-**	**-**
Challenge level	**0.05*** **	**-36.48*** **	**0.22*** **	**0.46*** **	**-0.23***
Response Rate	**-**	**-**	**-**	**-1.12*** **	**1.56*** **
Accurate RT	**-**	**-**	**-**	**0.61** **	**-0.71*** **
Mean Accuracy	**-**	**-**	**-**	**-1.13*** **	**1.51*** **
Goal Distance	**-**	**-27.88*** **	-0.01	**-**	**-**
**Experiment 2: Challenge level and Reward Level**					
Interval Goal Completion	**0.41*** **	**-171.30*** **	**6.24*** **	**-1.56*** **	**3.16*** **
Trial Goal Completion	**-**	**-33.26*** **	**-0.19*** **	**-**	-
Challenge level	**0.06*** **	**-37.03*** **	**1.34*** **	**0.25*** **	-0.06
Reward	**0.01*** **	**-4.60** **	1.01	**0.13*** **	**0.16*** **
Response Rate	-	-	-	**-0.88*** **	**1.32*** **
Accurate RT	-	-	-	**0.52*** **	**-0.76*** **
Mean Accuracy	-	-	-	**-0.70*** **	**1.06*** **
Goal Distance	-	**-20.38*** **	**1.14***	**-**	**-**

1. For effects of Challenge level, Reward, Performance on Affect, as well as Trial Goal Completion on RT and accuracy, the estimates reported are only of intervals where the goal had been reached.
Note: Values reported are beta estimates from linear mixed models, except for Accuracy, which are log odds ratios from binomial general linear models.
*p < .05, **p < .01, ***p < .001

Supplement Table 11. Mixed Model Results for Reaction Time Based on Goal Completion (Experiment 2)

Predictors	Before Goal Completion			After Goal Completion		
	Estimates	*SE*	*p-value*	*Estimates*	*SE*	*p-value*
Hard - Easy	-37.23	4.13	**< 0.001*** **	-26.78	4.06	**< 0.001*** **
High Reward – Low Reward	-4.61	1.74	**0.008** **	-6.95	2.28	**0.002** **
Interval Goal Completion	-170.35	15.96	**< 0.001*** **	/	/	/
Scaled Trial Session Num	-67.46	10.55	**< 0.001*** **	-57.75	13.20	**0.001*** **
Scaled Interval Session Num	54.12	10.09	**< 0.001*** **	46.41	13.40	**< 0.001*** **
Scaled Interval Block Num	-1.76	1.66	0.289	-3.14	1.99	0.115
Challenge X Reward	0.99	1.68	0.558	0.89	2.24	0.691

Supplement Table 12. Mixed Model Results for Trial Accuracy Based on Goal Completion (Experiment 2)

Predictors	Before Goal Completion			After Goal Completion		
	Log Odds Ratios	*SE*	*p-value*	*Log Odds Ratios*	*SE*	*p-value*
Hard - Easy	0.30	0.04	**< 0.001*** **	0.14	0.05	**0.009** **
High Reward – Low Reward	0.01	0.02	0.802	0.01	0.05	0.790
Interval Goal Completion	1.89	0.08	**< 0.001*** **	/	/	/
Scaled Trial Session Num	-0.73	0.14	**< 0.001*** **	-0.23	0.19	0.229
Scaled Interval Session Num	0.71	0.13	**< 0.001*** **	0.19	0.20	0.326
Scaled Interval Block Num	0.01	0.02	0.555	-0.01	0.03	0.822
Challenge X Reward	-0.02	0.02	0.420	-0.00	0.04	0.952

Supplement Table 13. Mixed Model Results for Goal Proximity Effect for Reaction Time and Accuracy (Experiment 2)

Predictors	Response Time (for accurate trials)			Accuracy		
	Estimates	*SE*	*p-value*	*Log Odds Ratios*	*SE*	*p-value*
Goal Distance	-20.38	1.91	**< 0.001*** **	0.13	0.05	**0.012***
Hard - Easy	-15.10	2.31	**< 0.001*** **	0.25	0.04	**< 0.001*** **
High Reward – Low Reward	-4.92	1.56	**0.002** **	0.02	0.03	0.622
Scaled Trial Session Num	-18.41	9.17	**0.045***	-0.69	0.16	**< 0.001*** **
Scaled Interval Session Num	6.94	9.11	0.446	0.69	0.16	**< 0.001*** **
Scaled Interval Block Num	-1.69	1.32	0.201	0.03	0.03	0.242
Goal Distance X Challenge	2.73	1.80	0.129	0.08	0.04	**0.028***

Goal Distance X Reward	-2.05	1.76	0.244	0.03	0.03	0.351
Challenge X Reward	0.70	1.54	0.650	0.03	0.03	0.306

Supplement Table 14. Mixed Model Results for Affective Ratings Based on Challenge Level, Reward, and Goal Completion (Experiment 2)

Predictors	Stress			Positive Affect		
	Estimates	*SE*	*p-value*	*Estimates*	*SE*	*p-value*
Hard - Easy	0.38	0.15	**0.015***	0.07	0.16	0.657
High Reward – Low Reward	0.08	0.14	0.550	-0.20	0.15	0.185
Interval Goal Completion	-1.56	0.27	**< 0.001*****	3.16	0.32	**< 0.001*****
Average Congruency	-0.02	0.03	0.646	0.05	0.03	0.180
Scaled Interval Session Num	-0.23	0.03	**< 0.001*****	-0.12	0.03	**< 0.001*****
Scaled Interval Block Num	0.04	0.03	0.202	-0.07	0.03	**0.046***
Challenge X Reward	0.01	0.14	0.958	0.09	0.15	0.537
Challenge X Interval Goal Completion	-0.13	0.15	0.393	-0.13	0.16	0.424
Reward X Interval Goal Completion	0.05	0.14	0.733	0.36	0.16	**0.022***

Supplement Table 15. Mixed Model Results for Affective Ratings Based on Response Rate (Goal Completed Intervals Only, Experiment 2)

Predictors	Stress			Positive Affect		
	Estimates	*SE*	*p-value*	*Estimates*	*SE*	*p-value*
Response Rate	-0.88	0.10	**< 0.001*****	1.32	0.14	**< 0.001*****
Hard - Easy	0.44	0.07	**< 0.001*****	-0.35	0.05	**< 0.001*****
High Reward – Low Reward	0.18	0.04	**< 0.001*****	0.10	0.04	**0.013***
Average Congruency	0.01	0.03	0.867	0.01	0.03	0.804
Scaled Interval Session Num	-0.14	0.03	**< 0.001*****	-0.21	0.03	**< 0.001*****
Scaled Interval Block Num	0.02	0.03	0.518	-0.06	0.03	**0.039***
Response Rate X Challenge	-0.09	0.06	0.127	0.17	0.05	**0.001*****
Response Rate X Reward	-0.02	0.05	0.694	-0.00	0.05	0.978
Challenge X Reward	0.08	0.04	**0.040***	-0.01	0.04	0.693

Supplement Table 16. Mixed Model Results for Affective Ratings Based on Average Reaction Time (Goal Completed Intervals Only, Experiment 2)

Predictors	Stress			Positive Affect		
	Estimates	*SE*	*p-value*	*Estimates*	*SE*	*p-value*
Mean Accurate RT	0.52	0.10	**< 0.001*****	-0.76	0.12	**< 0.001*****
Hard - Easy	0.34	0.06	**< 0.001*****	-0.15	0.05	**0.002****
High Reward – Low Reward	0.19	0.04	**< 0.001*****	0.15	0.05	**0.002****
Average Congruency	-0.00	0.04	0.998	0.03	0.04	0.445

Scaled Interval Session Num	-0.17	0.04	**< 0.001*** **	-0.19	0.04	**< 0.001*** **
Scaled Interval Block Num	0.02	0.03	0.648	-0.06	0.04	0.067
Mean Accurate RT X Challenge	0.09	0.06	0.146	-0.01	0.06	0.923
Mean Accurate RT X Reward	0.06	0.05	0.268	0.01	0.06	0.810
Challenge X Reward	0.10	0.04	**0.012***	-0.03	0.04	0.490

Supplement Table 17. Mixed Model Results for Affective Ratings Based on Average Accuracy (Goal Completed Intervals Only, Experiment 2)

Predictors	Stress			Positive Affect		
	Estimates	*SE*	*p-value*	*Estimates*	*SE*	*p-value*
Mean Accuracy	-0.70	0.07	**< 0.001*** **	1.06	0.11	**< 0.001*** **
Hard - Easy	0.32	0.06	**< 0.001*** **	-0.19	0.05	**< 0.001*** **
High Reward – Low Reward	0.16	0.04	**< 0.001*** **	0.13	0.04	**0.001*** **
Average Congruency	-0.02	0.04	0.602	0.02	0.03	0.488
Scaled Interval Session Num	-0.23	0.03	**< 0.001*** **	-0.12	0.03	**< 0.001*** **
Scaled Interval Block Num	0.03	0.03	0.417	-0.06	0.03	0.061
Mean Accuracy X Challenge	-0.01	0.06	0.916	0.14	0.06	**0.024***
Mean Accuracy X Reward	-0.08	0.06	0.181	0.02	0.06	0.725
Challenge X Reward	0.07	0.04	0.076	-0.01	0.04	0.725

Effects of affective experience and performance on subsequent task performance

We found that participants who had reached the goal on the previous interval reported feeling less stressed in the current interval ($F(1, 363.8) = 3.96$, p = 0.047, Supplement Table 20), which is directionally consistent with the non-significant trend we observed in Experiment 1. In contrast with Experiment 1's results, we found that participants who reported higher stress for the previous interval responded slower (not faster) in the current interval ($F(1, 65.3) = 5.41$, p = 0.020; Supplement Table 18). We also observed a trend for participants to feel higher levels of positive affect in the current interval when they had completed the previous goal ($F(1, 111.6) = 3.36$, p = 0.067), which is the opposite of the trend observed in Experiment 1. We did not find any other significant effects of previous interval affect on subsequent performance (See Supplement Table 18, Supplement Table 19). As in Experiment 1, meeting one's goal on the

previous interval predicted faster responding in the current interval (response rate: $F(1, 202.9) = 14.55$, $p < 0.001$, RT: $F(1, 94.2) = 16.54$, $p < 0.001$; Supplement Table 21).

Supplement Table 18. Mixed Model Results for Response Rate and Mean Accurate RT Based on Previous Interval's Stress Rating (Experiment 2)

Predictors	Response Rate			Mean Accurate RT		
	Estimates	*SE*	*p-value*	*Estimates*	*SE*	*p-value*
Previous Stress	-0.00	0.00	0.914	3.35	1.44	**0.020***
Hard - Easy	0.05	0.01	**< 0.001*****	-30.63	3.49	**< 0.001*****
High Reward – Low Reward	0.01	0.00	**0.012***	-3.78	2.19	0.085
Average Congruency	0.00	0.00	0.798	-3.11	2.22	0.161
Scaled Interval Session Num	0.01	0.00	**< 0.001*****	-8.53	2.18	**< 0.001*****
Scaled Interval Block Num	0.00	0.00	0.586	-3.06	2.52	0.225

Supplement Table 19. Mixed Model Results for Response Rate and Mean Accurate RT Based on Previous Interval's Positive Affect Rating (Experiment 2)

Predictors	Response Rate			Mean Accurate RT		
	Estimates	*SE*	*p-value*	*Estimates*	*SE*	*p-value*
Previous Positive Affect	0.00	0.00	0.343	-1.61	1.14	0.153
Hard - Easy	0.05	0.01	**< 0.001*****	-28.28	3.30	**< 0.001*****
High Reward – Low Reward	0.01	0.00	**0.002****	-6.69	2.29	**0.004****
Average Congruency	0.01	0.00	**0.009****	-6.58	2.23	**0.003****
Scaled Interval Session Num	0.03	0.00	**< 0.001*****	-13.68	2.12	**< 0.001*****
Scaled Interval Block Num	-0.00	0.00	0.427	1.21	2.36	0.609

Supplement Table 20. Mixed Model Results for Affective Ratings Based on Previous Interval's Goal Completion (Experiment 2)

Predictors	Stress			Positive Affect		
	Estimates	*SE*	*p-value*	*Estimates*	*SE*	*p-value*
Previous Interval Goal Completion	-0.31	0.15	**0.047***	0.28	0.15	0.067
Hard - Easy	0.20	0.06	**0.001*****	-0.01	0.05	0.838
High Reward – Low Reward	0.12	0.04	**0.008****	0.19	0.04	**< 0.001*****
Average Congruency	0.01	0.04	0.893	0.08	0.04	0.055*
Scaled Interval Session Num	-0.22	0.04	**< 0.001*****	-0.10	0.04	**0.010****
Scaled Interval Block Num	0.04	0.04	0.346	-0.05	0.04	0.249

Supplement Table 21. Mixed Model Results for Response Rate and Mean Accurate RT Based on Previous Interval's Goal Completion (Experiment 2)

Predictors	Response Rate			Mean Accurate RT		
	Estimates	*SE*	*p-value*	*Estimates*	*SE*	*p-value*
Previous Interval Goal Completion	0.04	0.01	**< 0.001***	-24.57	6.04	**< 0.001***
Hard - Easy	0.05	0.00	**< 0.001***	-30.51	3.09	**< 0.001***
High Reward – Low Reward	0.01	0.00	**< 0.001***	-4.84	1.55	**0.002**
Average Congruency	0.01	0.00	**0.031***	-5.08	1.56	**0.001***
Scaled Interval Session Num	0.02	0.00	**< 0.001***	-11.52	1.51	**< 0.001***
Scaled Interval Block Num	-0.00	0.00	0.764	-0.11	1.71	0.949
